# Stretched and compressed exponentials in the relaxation dynamics of a metallic glass-forming melt

**DOI:** 10.1038/s41467-018-07759-w

**Published:** 2018-12-17

**Authors:** Zhen Wei Wu, Walter Kob, Wei-Hua Wang, Limei Xu

**Affiliations:** 1 International Center for Quantum Materials, School of Physics, Peking University, 100871 Beijing, China; 2 School of Systems Science, Beijing Normal University, 100875 Beijing, China; 3 Laboratoire Charles Coulomb, University of Montpellier and CNRS, 34095 Montpellier, France; 4 Institute of Physics, Chinese Academy of Sciences, 100190 Beijing, China; 5 Collaborative Innovation Center of Quantum Matter, Beijing, China

## Abstract

The dynamics of glass-forming systems shows a multitude of features that are absent in normal liquids, such as non-exponential relaxation and a strong temperature-dependence of the relaxation time. Connecting these dynamic properties to the microscopic structure of the system is challenging because of the presence of the structural disorder. Here we use computer simulations of a metallic glass-former to establish such a connection. By probing the temperature and wave-vector dependence of the intermediate scattering function we find that the relaxation dynamics of the glassy melt is directly related to the local arrangement of icosahedral structures: Isolated icosahedra give rise to a liquid-like stretched exponential relaxation whereas clusters of icosahedra lead to a compressed exponential relaxation that is reminiscent to the one found in a solid. Our results show that in metallic glass-formers these two types of relaxation processes can coexist and give rise to a dynamics that is surprisingly complex.

## Abstract (web summary)

Glasses show peculiar relaxation dynamics below glass transition temperature, yet a deeper understanding of this phenomenon is still lacking. Wu et al. show the coexistence of stretched and compressed relaxation in a metallic glass system and attribute their origins to different local cluster structures.

## Introduction

The relaxation dynamics of glass-forming liquids has been and still is a topic of intensive research^1–3^. This activity is motivated by the fact that glasses are not only important for a multitude of industrial and daily applications but also pose a formidable intellectual challenge since so far there is no theoretical framework that is able to give a satisfactory description of the many unusual features of glass-forming liquids and glasses. For example, most glass-forming systems show in the liquid state a stretched exponential decay of their time correlation functions^4–6^, and it is believed that this is directly related to the so-called dynamical heterogeneities^4–7^ in the system, a phenomenon for which at present there is no solid theoretical understanding^8^. For temperatures slightly below the glass transition temperature *T*_g_, the systems show instead compressed exponentials that are speculated to be related to the release of internal stresses^9–14^, although also in this case we lack a good understanding for this behavior. Note that this type of stress relaxation is expected to be also important deep in the glass state^9,10^ and thus to be related to the mechanical properties of the material, hence giving a rational why certain glasses, such as metallic glasses, are ductile and others, for example, oxide glasses, are brittle^15,16^.

Since in glass physics all dynamical features are a smooth function of temperature, it must be expected that such internal stresses are in fact already present in the deeply supercooled melt and hence the compressed exponentials should be observable to some extent already at temperatures above *T*_g_, that is, in the glassy melt. However, detecting in experiments the coexistence of such different relaxation behavior is not an easy task, since one first needs to identify observables that are related to the different relaxation mechanisms and then be able to measure their corresponding correlation functions. Furthermore, it cannot be expected that all glass-forming systems will show such a coexistence and hence a wise choice on the material has to be made. Since for the case of metallic glasses one does indeed find a crossover from stretched to compressed relaxation if temperature is decreased^10,17^, such systems seem to be good candidates to detect the simultaneous presence of both relaxation mechanisms, and in the following we show that this expectation is indeed borne out.

For metallic glasses it is well documented that local icosahedral clusters are important structural building blocks^18,19^ and it has been found that atoms in these entities have a smaller than average atomic volume, a higher than average elastic modulus, and show a slower than average dynamics^20–23^. These observations indicate that these clusters are indeed relevant for the slowing down of the dynamics of the melt and the mechanical properties of the glass^24^. In the following, we will thus focus on atoms that are at the center of these icosahedra and in particular will consider various types of clusters formed by such interlocked icosahedra, that is, structures that have a size larger than the typical interatomic distance. By probing the wave-vector dependence of the relaxation of the intermediate scattering function for different clusters, we find that the relaxation dynamics shows a stretched exponential time dependence for weakly connected clusters, whereas strongly connected clusters have a compressed exponential time dependence, thus demonstrating that for metallic glasses these two types of processes can indeed coexist, a behavior that we expect to be related to the outstanding mechanical properties of these systems.

## Results

### Vibrational properties

**Fig. 1:**
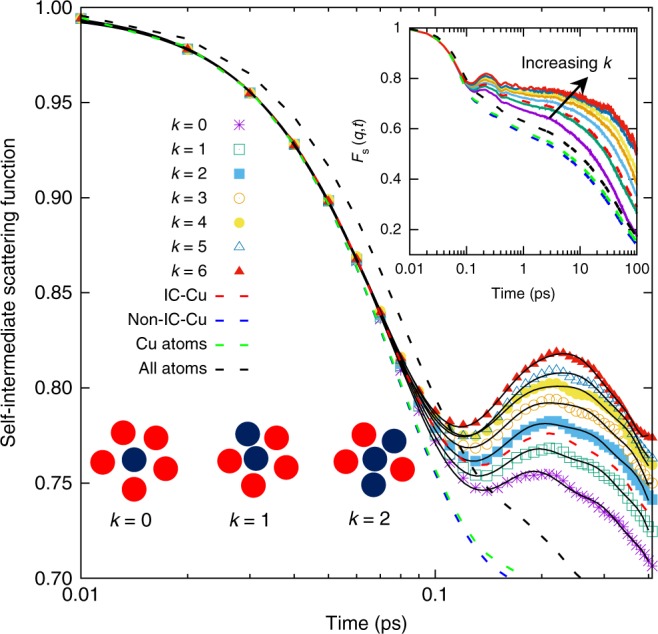
Relaxation dynamics of the system at short times. Short-time behavior of the self-intermediate scattering function of particles with different local connectivity *k* (symbols). The wave-vector is *q* = 2.8 Å^−1^ and *T* = 1000 K. With increasing *k* the height of the peak at around 0.2 ps increases showing that the motion becomes less damped. The solid lines are fits to the data with Eq. (2). Also included is *F*_s_(*q*, *t*) for the Cu atoms in an icosahedral cluster (dashed red line), the Cu atoms not in an icosahedral cluster (blue dashed line), and all Cu atoms (green). The black dashed line is the correlation function averaged over all atoms. The upper inset shows the same data in a larger time interval. The lower inset illustrates the definition of particles with different connectivities *k*: Particles in blue are the center of an icosahedral-like cluster

The studied Cu_50_Zr_50_ system consists of *N* = 10,000 atoms interacting via an embedded atom potential^25^. (See Methods for details on the simulations.) For this system the onset temperature, that is, the point at which the dynamics becomes glassy^2^, is around 1250 K (see Supplementary Information), and in the following we study the properties of the system between 950 and 1100 K, that is, in a *T-*range in which the dynamics is already rather glassy. (See Supplementary Information for the *T*-dependence of the relaxation time.) As discussed above, in this system icosahedral-like clusters with a Cu atom in its center constitute slowly relaxing structures^18,21,23^ and hence we focus here on the dynamics of these entities. For this we use a Voronoi construction^24,26^ to identify those Cu atoms that are exactly 12-fold coordinated, that is, are in the center of an icosahedron. For each of these atoms, we count the number, *k*, of neighboring Cu atoms that are also 12-fold coordinated and we refer the central Cu atom to be in the center of a *k*-cluster (see lower inset of Fig. 1)^21,27^. (When we discuss in the following the dynamic properties of a *k*-clusters we refer to the value of *k* at time zero.) In the Supplementary Information, we show how the concentration of these populations change with temperature *T*. The space and time dependence of the relaxation dynamics can be characterized by means of the self-intermediate scattering function (SISF) *F*_s_(*q*, *t*), where *q* is the wave-vector:

1 $$F_{\mathrm{s}}(q,t) = \frac{1}{N}\mathop {\sum}\limits_{j = 1}^N \left\langle {{\mathrm{exp}}\left[ { - {\mathrm{i}}{\bf{q}} \cdot \left( {{\bf{r}}_j(t) - {\bf{r}}_j(0)} \right)} \right]} \right\rangle .$$

Here *N* is the number of particles considered, 〈.〉 is the thermal average, and **r**_*j*_(*t*) is the position of particle *j* at time *t*. In Fig. 1 we show the time dependence of *F*_s_(*q*, *t*) for particles that have different connectivity. The wave-vector is 2.8 Å^−1^ which corresponds to the main peak in the static structure factor (see Supplementary Fig. 2 in the Supplementary Information). We recognize that for *t* ≤ 0.1 ps the different curves fall on a common curve, showing that on this time scale the motion of the particles is independent of their environment, that is, of *k*, since up to this time the motion is ballistic^21^. This is no longer the case for somewhat larger *t* in that the correlators for large *k* show a marked peak at around 0.2 ps. The presence of such a peak indicates that on this time scale the motion of large-*k* particles has the character of a damped oscillator, and in the Supplementary Information we show that this feature is indeed also very pronounced in the glass state (see Supplementary Fig. 9). This *k-*dependence of *F*_s_(*q*, *t*) shows that with increasing *k* the cage becomes stiffer and the damping is weaker. In previous studies the presence of such a peak has been associated with the so-called boson peak^28–32^, that is, an excess in the vibrational density of states (DOS) in the glass state^2^. Such an interpretation is indeed compatible for the present system as well, since we do find a peak at around 4 THz if one divides the vibrational DO S by the square of the frequency, see Supplementary Fig. 3b in the Supplementary Information, which matches well the 0.2 ps found for the peak in *F*_s_(*q*, *t*). Also included in the graph is the correlator for the Cu atoms that are not in the center of an icosahedron and we see that this function decays significantly faster than the one for the icosahedrally packed atoms (also shown), in agreement with the results from ref. ^23^. Furthermore, we see that the former correlator shows no peak at intermediate times, thus confirming that this peak is related to the highly structured local environment of the central atom. The upper inset in the figure shows the same correlators on a much larger time range, demonstrating that the *k*-dependence of the SISF is present even on the time scale of the *α*-relaxation, see below for more details, thus indicating a connection between the dynamics at short times with the one at long times^33–35^.

**Fig. 2:**
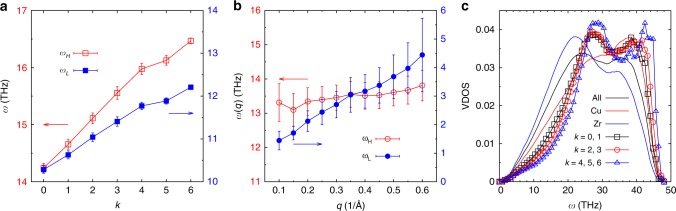
The dependence of the vibrational features on connectivity *k*. **a** The frequency of the high- and low-frequency modes, *ω*_*H*_ and *ω*_*L*_, increases with increasing *k*. *q* = 2.8 Å^−1^. **b** The frequency *ω*_*H*_, averaged over all *k* values, is basically independent of the wave-vector *q* showing that this is an optical mode, whereas the low-frequency mode at *ω*_*L*_(*q*) increases linearly with *q*, characteristic of an acoustic mode. Error bars in **a**, **b** have been obtained from the fit of the SISF with Eq. (2). **c** Vibrational density of states for the different populations showing that the highly connected icosahedra have on average higher frequencies

To describe the dynamics at these short times in a quantitative manner, we fit the correlator with the simple Ansatz

2 $$F_{\mathrm{s}}(q,t) = \mathop {\sum}\limits_{j = L,H} {\kern 1pt} C_j{\kern 1pt} {\mathrm{exp}}\left\{ {{\mathrm{i}}q\left[ {A{\kern 1pt} {\mathrm{cos}}\left( {\omega _jt + \delta _j} \right) - A{\kern 1pt} {\mathrm{cos}}\left( {\delta _j} \right)} \right]} \right\},$$

where *L* and *H* denote a low- and high-frequency mode, respectively, and we have *C*_*L*_ + *C*_*H*_ = 1, that is, we describe the motion of a particle as a superposition of two harmonic oscillators. The resulting fits are included in Fig. 1 as well and we see that this functional form gives indeed a good description of the data. Figure 2a presents the *k*-dependence of *ω*_*H*_ and *ω*_*L*_ and one recognizes that both of them increase with *k* (basically linearly, with a small bend at around *k* = 5), which demonstrates that with increasing connectivity the cage becomes stiffer. Figure 2b shows the *q*-dependence of the two frequencies at low *q* and one sees that *ω*_*L*_ is a linear function of *q*, as expected for a generic SISF that couples at low *q* to the acoustic modes^36^. (For this plot we have averaged over all values of *k* since the *k*-dependence seen in panel a depends only weakly on *q*.) In contrast to this, *ω*_*H*_ is independent of *q*, showing that this mode has an optical character. In view of the observed *k*-dependence seen in Fig. 2, we can conclude that the peak in *F*_s_(*q*, *t*) at around 0.2 ps is directly related to the details of the local structure. Similar results are found for the coherent intermediate scattering function.

In order to put *ω*_*H*_ and *ω*_*L*_ in relation with typical frequencies in the system, we show in Fig. 2c its DOS at 0 K as obtained for the whole system and the partial densities of states for the two types of atoms, as well as for the Cu atoms having different connectivities (see Methods). One sees that with increasing *k* the peaks at around 28 and 42 THz increase significantly, that the upper limit of the DOS moves to higher frequencies, and that the DOS between 10 and 20 THz is lowered. Thus, this *k*-dependence of the DOS is coherent with the result from Fig. 2a that the stiffness of the cage increases with increasing connectivity of the local structure.

### Relaxation dynamics

**Fig. 3:**
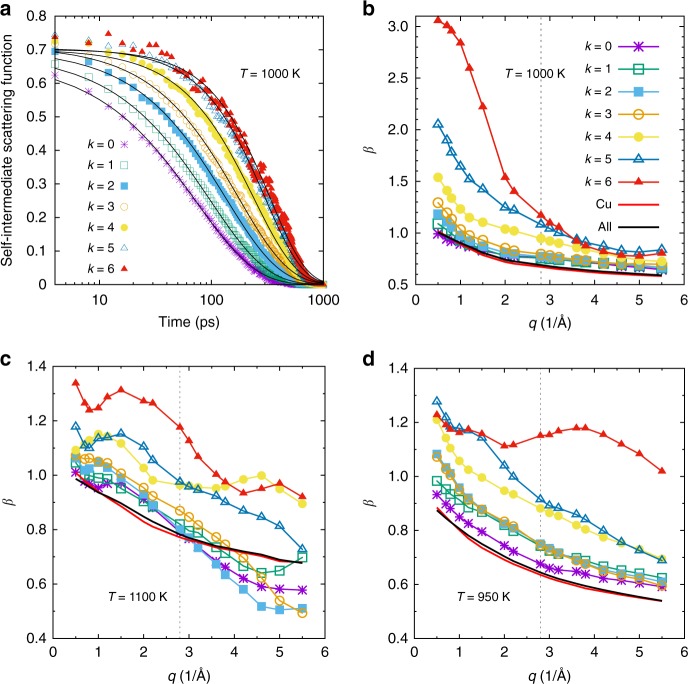
Relaxation dynamics of the system at intermediate and long times. **a** Self-intermediate scattering function at wave-vector *q* = 2.8 Å^−1^ for different values of connectivity *k* of the Cu atoms. *T* = 1000 K. **b** *q-*dependence of the KWW exponent *β* for different values of *k*. Also included is the data obtained if one averages over all Cu atoms and all atoms at *T* = 1000 K. **c**, **d** show *β* for temperatures *T* = 1100 K and 50 K, respectively. The vertical dashed line in **b**–**d** shows the position of the main peak in the static structure factor

To connect this vibrational motion at short times with the one at large *t*, we have fitted the SISF for the populations with different *k* with a Kohlrausch–Williams–Watts (KWW) function, that is, *F*_s_(*q*, *t*) = *A* exp(−(*t*/*τ*)^*β*^), where the prefactor *A*, the relaxation time *τ*, and the stretching exponent *β* depend on *q*. Figure 3a displays these data and the fits and one sees that the correlator has in the *α*-regime a very strong *k*-dependence. Figure 3b presents the *q*-dependence of the KWW exponent *β* for the different values of *k*. One recognizes that for small *k*, that is, isolated icosahedral clusters, *β* is smaller than 1.0 for all wave-vectors considered, that is, the relaxation is stretched as one expects for a glass-forming system^2^. Interestingly, we find that for intermediate and small *q* the exponent increases significantly with *k* and becomes larger than 1.0, that is, one sees a crossover from a normal glassy dynamics to one in which the correlator has a much sharper decay in time, and that this crossover depends strongly on *q*, that is, the length scale considered. The quick decay of the correlation function for large *k* indicates a sudden yielding of the structure, that is, a type of motion that is very different from the viscous flow found in glassy systems. The comparison of panel b with panels c and d of that figure shows that for large *k* the *q*-dependence of *β* is much more pronounced for *T* = 1000 K than for the two other neighboring temperatures and also that the maximum attained value of *β* is significantly larger. Thus, this observation shows that for temperature around 1000 K, there is a strong dependence of the nature of the relaxation dynamics for large-*k* clusters.

**Fig. 4:**
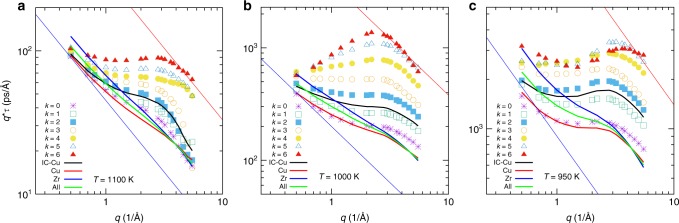
Wave-vector dependence of the *α-*relaxation time. We plot *τ*(*q*)*q* as a function of *q* for different values of the connectivity *k*. Also included is the data for all Cu/Zr atoms and all atoms. The three panels correspond to different temperatures: **a** *T* = 1100 K, **b** *T* = 1000 K, and **c** *T* = 950 K. The solid lines are power-laws with exponent −1, the *q* dependence expected for a diffusive process

To understand the nature of this abrupt yielding, we look at the *q*-dependence of the *α*-relaxation time *τ* as obtained from the fit with the KWW form. For a diffusive motion one expects that *τ* ∝ *q*^−2^, whereas for a ballistic displacement one has *τ* ∝ *q*^−1^. To allow for a better distinction of these two cases, we plot in Fig. 4 the product *τ*(*q*) · *q* as a function of *q*. The data show that at high *T*, panel a, the *q*-dependence of *τ* is close to *q*^−2^ if *k* is small, that is, the dynamics is diffusive, as expected for a supercooled liquid at intermediate and small *q*. For large *k* we find, however, a plateau in the *q*-dependence, indicating that on these length scales the relaxation dynamics is ballistic, that is, the icosahedron moves like a rigid structure. This shows that icosahedra which have in their first nearest neighbor shell many Cu atoms that are themselves at the center of an icosahedron are sufficiently rigid to be convected by the surrounding medium which is formed by low-*k* Cu atoms and Zr atoms.

If *T* is lowered to 1000 K, panel b, we find that now also the curves for small and intermediate *k* show a plateau, that is, at this temperature also these icosahedra have become sufficiently rigid to be moved as a rigid block. For the icosahedra with large *k* one recognizes that *τ* decays somewhat slower than *q*^−1^, which implies a relaxation that is faster than a rigid translation dynamics. We see that for the largest *k*’s this rapid relaxation dynamics is observed in a range 0.5 Å^−1^ ≤ *q* ≤ 2 Å^−1^, that is, it extends from about one interparticle distance up to distances that are on the order of the size of a few icosahedra, thus about 1 nm. The fact that on these length scales the relaxation time is almost constant indicates that the underlying structure breaks up in a very abrupt manner. In the Supplementary Information, Supplementary Fig. 5, we show that clusters with high *k* have a life time that is significantly longer than the one with small *k*, but that once the former start to disintegrate they do this very quickly, whereas clusters with small *k* decay in a more continuous manner. This rapid breaking up hints therefore to a yielding process that is more similar to the one found in a solid that fractures than to the continuous transformation found in a liquid. Since high *k* clusters have a longer life time than the ones with small *k* and move significantly slower than the latter, Fig. 4, we can conclude that the high *k* icosahedra form rigid structures that are moved by the surrounding viscous medium, which in turn is driven by local internal stresses.

Comparing these data with the one at *T* = 1100 K shows that the decrease of *T* affects more strongly the relaxation dynamics for the icosahedra with high *k* than the ones with low *k*. This implies that for this system, the so-called *α*-scale universality predicted by mode-coupling theory^37^ does not hold, that is, the relaxation time *τ*_*x*_(*T*) for an arbitrary observable *x* cannot be written as *τ*_*x*_(*T*) = *h*_*x*_*f*(*T*), where *h*_*x*_ is a *x*-dependent constant and *f*(*T*) is a system universal function of temperature. (In our case *x* can be *q* or *k*.) The fact that the *α*-scale universality does not hold for the present system, whereas it works for simple glass-forming systems such as binary Lennard–Jones mixtures^38^, shows that metallic glass formers have a surprisingly complex local dynamics and hence mean-field-like theories are not able to catch these features of the relaxation.

If the temperature is lowered even more, see data for *T* = 950 K in panel c, the unusual *q*-dependence found for large *k* is less pronounced and the relaxation dynamics shows again a stronger *q*-dependence and becomes similar to the one found at 1100 K. At the same time, the curves for low *k* show a plateau that is more pronounced than the one at higher *T*s, which makes that on overall the *k*-dependence of *τ* becomes weaker, that is, the relaxation dynamics of the system becomes more homogeneous. The likely reason for these changes is that a decrease in temperature will lead to high-*k* icosahedra that are somewhat more rigid but that the viscosity, which is roughly proportional to *τ*, increases significantly. Hence, the shear forces acting on the icosahedra will have increased substantially, making the latter to break up in a more continuous manner and thus leading to the disappearance of the faster than ballistic regime in *τ*(*q*). Thus, we can conclude that the different mechanical properties of the icosahedra with different *k* make that the nature of their relaxation dynamics depends strongly on *k* as well as temperature.

Also included in the graphs are the relaxation times as obtained for all the Cu atoms that are in the center of an icosahedron (black solid line) and one sees that the discussed change in transport mechanism makes that the *q*-dependence of *τ* for this population leads to the formation of a small shoulder at 1000 K and a small hump at 950 K. If *all* Cu atoms are considered (red line), one finds at high and intermediate temperatures only a weak shoulder and at the lowest temperature a pronounced plateau. In contrast to this, the Zr atoms have at high and intermediate temperatures a *q*-dependence that is close to the one for a diffusive dynamics and only at the lowest temperature one can notice a weak shoulder (blue line). The same is true if one looks at the *τ*(*q*)·*q* when averaged over all atoms (solid green line). These results thus demonstrate that the described anomalous behavior in the relaxation dynamics is detectable also in real experiments that can only measure the system-averaged quantities.

So far, we have discussed the relaxation dynamics of the particles in reciprocal space. Since in simulations one can also easily access the trajectories of the particles in real space, we can use this information to check the interpretation of the *q*-space data. For this, we have determined the self part of the van Hove function defined as^39^

3 $$G_{\mathrm{s}}(r,t) = \frac{1}{N}\mathop {\sum}\limits_{i = 1}^N \left\langle {\delta \left( {r - \left| {{\bf{r}}_i(0) - {\bf{r}}_i(t)} \right|} \right)} \right\rangle .$$

**Fig. 5:**
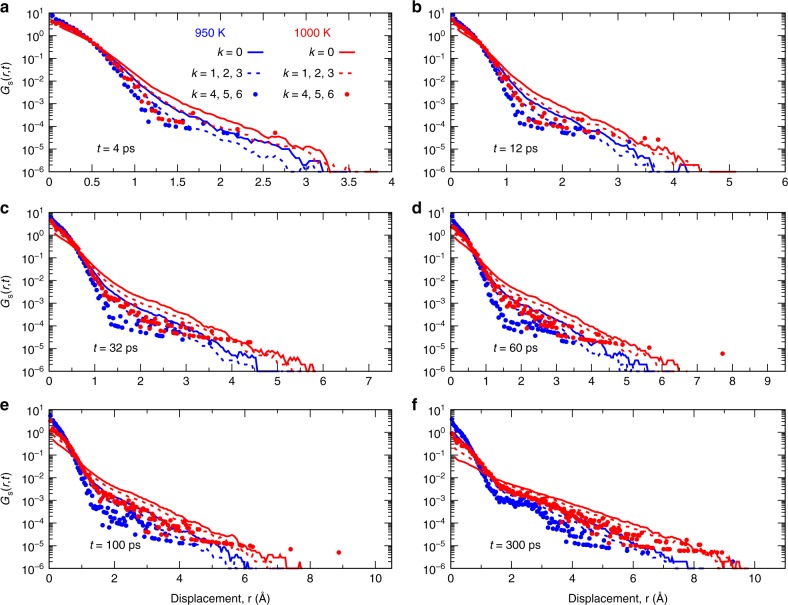
Space dependence of the self part of the van Hove correlation function. The curves correspond to *k* = 0 (full lines), the average over *k* = 1, 2, 3 (dashed lines), and the average over *k* = 4, 5, 6 (dotted lines). The red and blue curves are for *T* = 1000 K and 950 K, respectively. The panels correspond to different times *t* (given in the panels)

This distribution is shown in Fig. 5 for different times *t* and values of *k*. One recognizes that for *k* = 0 and short times *G*_s_(*r*, *t*) is given by a Gaussian at short distances and by an exponential tail at large *r*, that is, the typical behavior found in glassy liquids^40^. At intermediate times, *t* ~ 12–32 ps, we find that for *k* > 0 the distribution at distances larger than *r* ≈ 1.5 Å has a much smaller slope than the one for *k* = 0, which shows that particles with high *k* explore these large distances very rapidly, in agreement with the results shown in Fig. 4. For large times, panel f, the distribution for the different *k* become similar to each other at large *r*, while they differ significantly for *r* ≲ 1.5 Å in that *G*_s_(*r*, *t*) still has a Gaussian shape if *k* is large, whereas it is exponential like for small *k*. Since we find that on this time scale the clusters with large *k* start to loose their identity (see Supplementary Fig. 5), we can conclude from *G*_s_(*r*, *t*) that the particles which are still in the original environment do rattle around in their cage, thus giving rise to the Gaussian distribution, whereas those particles that have left their original cage start to move more quickly and hence have a distribution that is similar to the one found for the particles in low *k* clusters.

We also mention that since the mean-squared displacement (MSD) is just the second moment of *G*_s_(*r*, *t*), this *k*-dependence is also seen in the time dependence of the MSD (see Supplementary Fig. 4): for *T* = 1000 K the MSD shows for large *k* a jump-like increase (see Supplementary Information), a *t*-dependence that is again very different from the one found in usual glass-forming liquids^2^, while for the other temperatures the time dependence is very similar to the one of standard glass-forming systems.

## Discussion

Our results show that even in the fluid state certain glassy systems can show a relaxation dynamics that is given by a compressed exponential, that is, a time dependence that differs strongly from the usual stretched exponential found in viscous liquids. For the metallic glass former investigated here, these two types of relaxation dynamics do even coexist and can be directly related to the local atomic structure. Although the different relaxation behavior is most easily seen if one considers structural entities that are beyond the atomic distances, we find that a careful analysis of the wave-vector dependence of the relaxation times of the particle averaged *F*_s_(*q*, *t*) does show the fingerprint of the two relaxation mechanisms. The relative importance of these mechanisms depends strongly on temperature as well as the length scale considered, that is, the wave-vector, and becomes very pronounced at around the mode-coupling temperature of the system, and gives rise to a decoupling phenomena in the dynamics that is non-monotonic in temperature. It can be speculated that the structural features that give rise to these different relaxation mechanisms in the liquid are also responsible for the interesting mechanical properties of metallic glasses, such as elasticity and ductility, in that the more rigid structures formed by the high-*k* icosahedra are embedded by a softer surrounding that allows for localized plastic relaxation. A detailed study that probes the existence of such a connection would certainly be very valuable. Thus, our findings show that a careful investigation of the wave-vector dependence of the relaxation dynamics allows to get deeper insight into the unique properties of metallic glasses, insight that will help to conceive other new materials that do have attractive mechanical properties.

## Methods

### Potential and simulation

The interaction potential used is the one proposed by Mendelev et al.^25^ and http://www.ctcms.nist.gov/potentials/Cu-Zr.html, which has been found to be one of the most reliable embedded atom potentials for describing Cu-Zr systems^41^. Simulations have been carried out using the LAMMPS software^42^. The time step was 1 fs and the initial atomic configuration was firstly equilibrated for long time (2 ns) at temperature *T* = 2000 K in the NPT ensemble (*P* = 0 bar) using a Nose–Hoover thermostat and barostat. The liquid was then cooled down to its target temperature at a rate of 1 K/ps at a constant pressure *P* = 0 and subsequently relaxed for 1 ns before the structure and dynamics data were collected.

### Density of states

The vibrational DOS was calculated by making a run at low temperatures and calculating the time Fourier transform of the velocity-autocorrelation function of the species of interest, that is, Cu and Zr. We have checked that this approach gives the same results as a direct diagonalization of the dynamical matrix. See Supplementary Information for a comparison between the two methods.

### Reporting summary:

Nature Communications thanks the anonymous reviewer(s) for their contribution to the peer review of this work.

## Supplementary information

Supplementary Information

## Data Availability

The datasets generated during and/or analyzed during the current study are available from the corresponding author on reasonable request.

## References

[1] Debenedetti PG, Stillinger FH (2001). Supercooled liquids and the glass transition. Nature.

[2] Binder K, Kob W (2011). Glassy Materials and Disordered Solids: An Introduction to Their Statistical Mechanics.

[3] Varshneya AK (2006). Fundamentals of Inorganic Glasses.

[4] Ediger MD, Angell CA, Nagel SR (1996). Supercooled liquids and glasses. J. Phys. Chem..

[5] Kob W, Andersen HC (1995). Testing mode-coupling theory for a supercooled binary Lennard–Jones mixture. II. Intermediate scattering function and dynamic susceptibility. Phys. Rev. E.

[6] Kob W (1999). Computer simulations of supercooled liquids and glasses. J. Phys..

[7] Puosi F, Jakse N, Pasturel A (2018). Dynamical, structural and chemical heterogeneities in a binary metallic glass-forming liquid. J. Phys.

[8] Berthier, L., Biroli, G., Bouchaud, J.-P., Cipelletti, L. & van Saarloos, W. (eds).*Dynamical Heterogeneities in Glasses, Colloids and Granular Materials* (Oxford University Press, Oxford, 2011).

[9] Ruta B (2014). Revealing the fast atomic motion of network glasses. Nat. Commun..

[10] Ruta B (2012). Atomic-scale relaxation dynamics and aging in a metallic glass probed by X-ray photon correlation spectroscopy. Phys. Rev. Lett..

[11] Cipelletti L, Manley S, Ball RC, Weitz DA (2000). Universal aging features in the restructuring of fractal colloidal gels. Phys. Rev. Lett..

[12] Ballesta P, Duri A, Cipelletti L (2008). Unexpected drop of dynamical heterogeneities in colloidal suspensions approaching the jamming transition. Nat. Phys..

[13] Caronna C, Chushkin Y, Madsen A, Cupane A (2008). Dynamics of nanoparticles in a supercooled liquid. Phys. Rev. Lett..

[14] Guo H (2009). Nanoparticle motion within glassy polymer melts. Phys. Rev. Lett..

[15] Matteo C (2009). Stress-corrosion mechanisms in silicate glasses. J. Phys. D.

[16] Sun BA, Wang WH (2015). The fracture of bulk metallic glasses. Prog. Mater. Sci..

[17] Luo P, Wen P, Bai HY, Ruta B, Wang WH (2017). Relaxation decoupling in metallic glasses at low temperatures. Phys. Rev. Lett..

[18] Lad KN, Jakse N, Pasturel A (2012). Signatures of fragile-to-strong transition in a binary metallic glass-forming liquid. J. Chem. Phys..

[19] Jaiswal A, Egami T, Zhang Y (2015). Atomic-scale dynamics of a model glass-forming metallic liquid: dynamical crossover, dynamical decoupling, and dynamical clustering. Phys. Rev. B.

[20] Wakeda M, Shibutani Y (2010). Icosahedral clustering with medium-range order and local elastic properties of amorphous metals. Acta Mater..

[21] Wu ZW, Li MZ, Wang WH, Liu KX (2013). Correlation between structural relaxation and connectivity of icosahedral clusters in CuZr metallic glass-forming liquids. Phys. Rev. B.

[22] Li M, Wang CZ, Hao SG, Kramer MJ, Ho KM (2009). Structural heterogeneity and medium-range order in Zr_*x*_Cu_100*−x*_ metallic glasses. Phys. Rev. B.

[23] Li FX, Li MZ (2017). Local environments of atomic clusters and the effect on dynamics in CuZr metallic glass-forming liquids. J. Appl. Phys..

[24] Sheng HW, Luo WK, Alamgir FM, Bai JM, Ma E (2006). Atomic packing and short-to-medium-range order in metallic glasses. Nature.

[25] Mendelev MI, Sordelet DJ, Kramer MJ (2007). Using atomistic computer simulations to analyze x-ray diffraction data from metallic glasses. J. Appl. Phys..

[26] Finney JL (1977). Modelling the structures of amorphous metals and alloys. Nature.

[27] Wu ZW (2016). Critical scaling of icosahedral medium-range order in CuZr metallic glass-forming liquids. Sci. Rep..

[28] Angell CA (1995). Formation of glasses from liquids and biopolymers. Science.

[29] Horbach J, Kob W, Binder K, Angell CA (1996). Finite size effects in simulations of glass dynamics. Phys. Rev. E.

[30] Kob W, Barrat JL (1997). Aging effects in a Lennard–Jones glass. Phys. Rev. Lett..

[31] Horbach J, Kob W, Binder K (2001). High frequency sound and the boson peak in amorphous silica. Eur. Phys. J. B.

[32] Sastry S, Austen Angell C (2003). Liquid–liquid phase transition in supercooled silicon. Nat. Mater..

[33] Scopigno T, Ruocco G, Sette F, Monaco G (2003). Is the fragility of a liquid embedded in the properties of its glass?. Science.

[34] Luo P, Li YZ, Bai HY, Wen P, Wang WH (2016). Memory effect manifested by a boson peak in metallic glass. Phys. Rev. Lett..

[35] Shintani H, Tanaka H (2008). Universal link between the boson peak and transverse phonons in glass. Nat. Mater..

[36] Sette F, Krisch MH, Masciovecchio C, Ruocco G, Monaco G (1998). Dynamics of glasses and glass-forming liquids studied by inelastic X-ray scattering. Science.

[37] Götze W (2008). Complex Dynamics of Glass-forming Liquids: A Mode-Coupling Theory.

[38] Gleim T, Kob W (2000). The *β*-relaxation dynamics of a simple liquid. Eur. Phys. J. B.

[39] Hansen JP, McDonald IR (2006). Theory of Simple Liquids.

[40] Chaudhuri P, Berthier L, Kob W (2007). Universal nature of particle displacements close to glass and jamming transitions. Phys. Rev. Lett..

[41] Lad KN, Jakse N, Pasturel A (2017). How closely do many-body potentials describe the structure and dynamics of Cu–Zr glass-forming alloy?. J. Chem. Phys..

[42] Plimpton S (1995). Fast parallel algorithms for short-range molecular dynamics. J. Comput. Phys..

