# Supplementary material for: Stretched and compressed exponentials in the relaxation dynamics of a metallic glass-forming melt
Source: Nat Commun. 2018 Dec 17;9:5334. doi: 10.1038/s41467-018-07759-w (PMC6297352; doi:10.1038/s41467-018-07759-w)
Supplement: Supplementary file 1 — Supplementary Information [file 41467_2018_7759_MOESM1_ESM.pdf]

# Supplementary Information
# Stretched and compressed exponentials in the relaxation dynamics of a metallic glass-forming melt

**Zhen Wei Wu, Walter Kob, Wei-Hua Wang, and Limei Xu**

**Supplementary Note 1**: Icosahedra

In Supplementary Figure 1 we show the probability that an icosahedron with a Cu atom at its center has connectivity $k$. We see that at high temperatures most icosahedra are of type $k = 0$ and that with decreasing temperature the icosahedra become increasingly connected.

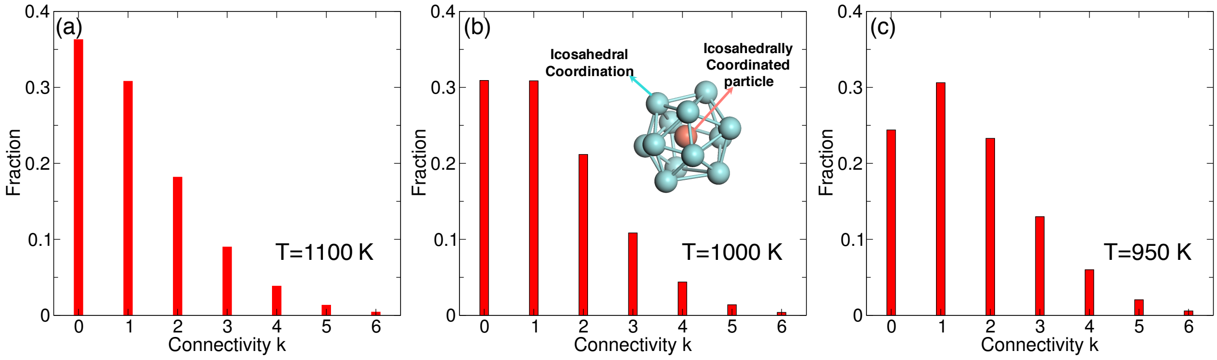


Supplementary Figure 1. Probability that an icosahedron is of type $k$. (a) $T = 1100$ K, (b) $T = 1000$ K, (c) $T = 950$ K. The inset in panel (b) shows a snapshot with a Cu at the center of an icosahedron.

**Supplementary Note 2**: Static structure factor

Supplementary Figure 2 shows the $q-$dependence of the partial static structure factors at three different temperatures. One recognizes that these functions show basically no $T-$dependence. The main peak for the Cu-Cu correlation is around 2.8 Å$^{-1}$, the wave-vector we often focus on in the present study.

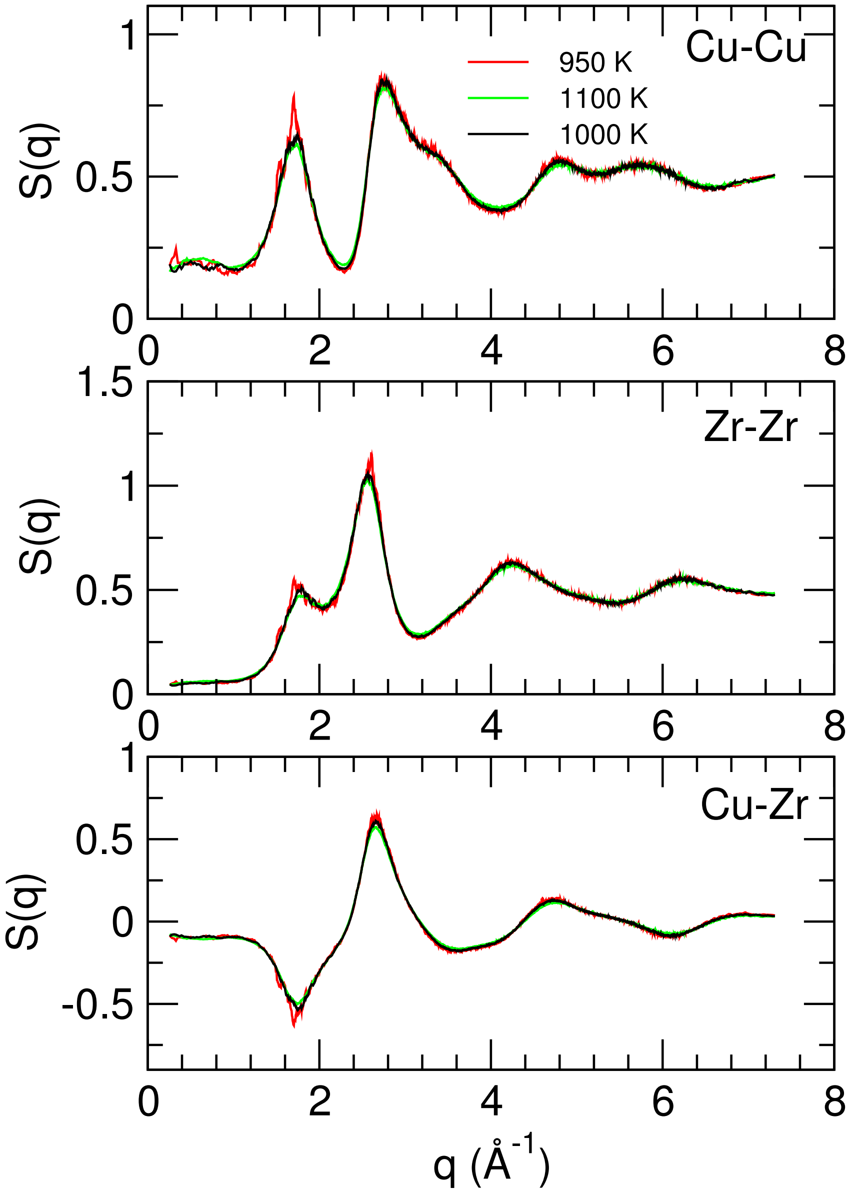


Supplementary Figure 2. $q-$dependence of the partial structure factors for the three temperatures considered.

**Supplementary Note 3**: Vibrational density of states

Supplementary Figure 3(a) shows the vibrational density of states (VDOS) $D(\omega)$ for two system sizes. For $N = 10^5$ particles we have computed $D(\omega)$ by calculating the time-Fourier transform of the velocity autocorrelation function of the particles. The data for $T = 300$ K agrees very well with the one obtained at $T = 10$ K, indicating that we are indeed probing the harmonic regime. For the smaller system size with $N = 10^4$ particles we have obtained $D(\omega)$ by diagonalizing directly the Hessian matrix evaluated at a local minimum of the potential energy of the system. The so obtained VDOS agrees well with the one obtained for the larger system.

In Supplementary Figure 3(b) we show the vibrational density of states divided by $\omega^2$. This type of plot can be used to probe for the existence of a boson peak in the system. The data shows that there is indeed evidence for the presence of a peak at around 4 THz which can be interpreted as a boson peak.

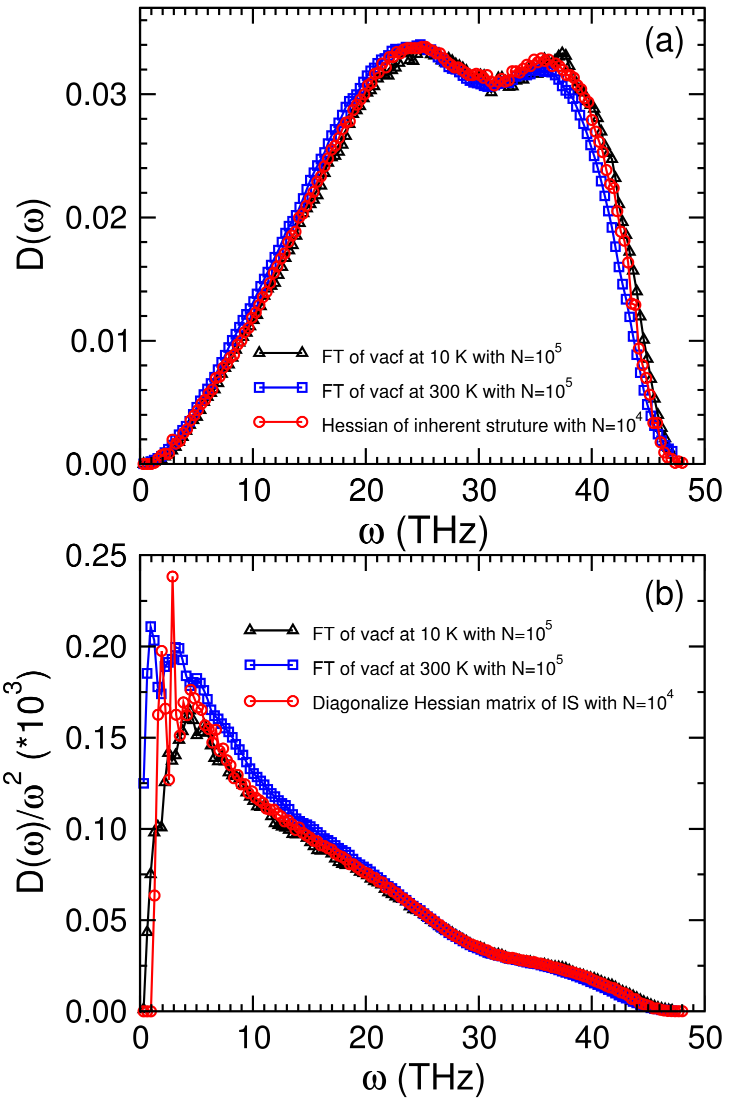


Supplementary Figure 3. (a) Vibrational density of states for $N = 10^4$ particles and $N = 10^5$ particles. For the system with $N = 10^4$ particles we have obtained the VDOS by the direct diagonalization of the Hessian matrix and for the $N = 10^5$ system by the calculation of the time Fourier transform of the velocity auto-correlation function. (b) VDOS/$\omega^2$ to test for the presence of a boson peak.

**Supplementary Note 4**: Mean squared displacement

In Supplementary Figure 4 we show the time dependence of the mean squared displacement (MSD) of various species: Cu atoms, Cu atoms inside an icosahedron, Cu atoms inside an icosahedron of connectivity $k$, and average over all atoms. One sees that the IC-Cu atoms move slower that the average Cu atom which in turns move a bit faster than the Zr atoms, results that are in agreement with previous studies. For $T = 1100$ K the $k-$dependence of the MSD is relatively weak and all the curves are smooth. For $T = 1000$ K there is a more pronounced $k-$dependence of the MSD and the data for large $k$ shows a rapid variation on the time scale of 50-200 ps. These jumps are an indication that in this time regime the particles undergo a rapid motion, compatible with the view expressed in the main text that these high$-k$ structures break up in a very sudden manner. If $T$ is lowered to 950 K, these jumps are no longer visible, in agreement with the discussion in the main text regarding the shape of the time correlation functions.

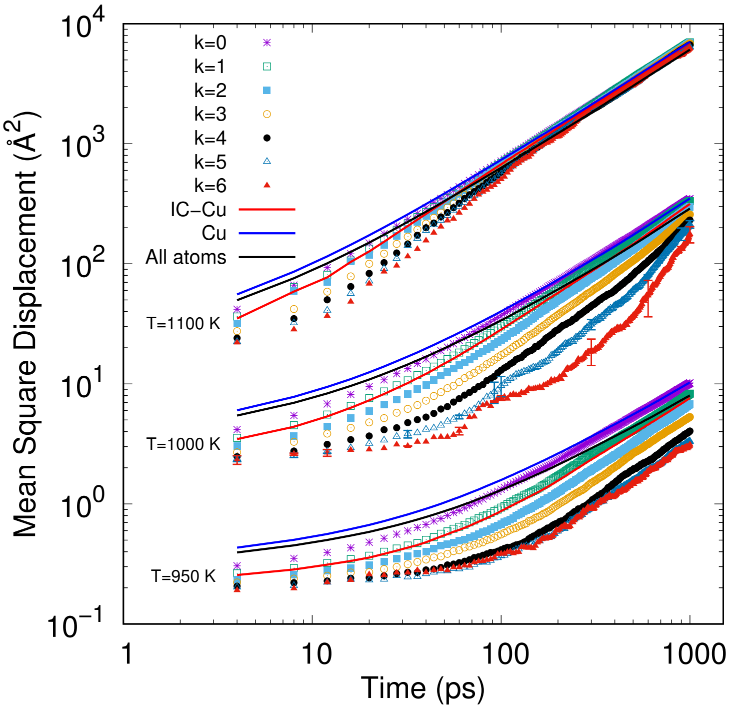


Supplementary Figure 4. Mean squared displacement for different type of atoms for three different temperatures. Cu: Any Cu atoms; IC-Cu: Any Cu that is at the center of an icosahedron; the data labeled with $k$ are the MSD for Cu with connectivity $k$. For $T = 1100$ K and $T = 1000$ K the curves have been shifted upward by a factor of 10 and 100, respectively.

**Supplementary Note 5**: Life time of clusters

In the main text we have shown that the clusters with large $k$ have a relaxation time that is significantly larger than the one of the clusters with small $k$, i.e., the former clusters move slower than the latter ones. In order to investigate the life time of the clusters we have determined the probability that a $k-$cluster which was present at time zero is still present at time $t$, i.e., that none of the neighbors of the central Cu atoms has been exchanged [1]. This probability is shown in Supplementary Figure 5 for the three temperatures investigated. We see that at the highest $T$ the curves for the different values of $k$ are relatively close together, i.e., the life time of the clusters does not strongly depend on $k$. For $T = 1000$ K this changes in that the clusters with high $k$ live about 10 times longer than the ones with small $k$. Furthermore also the shape of the curves depends strongly on $k$ in that the weakly connected clusters loose their identity in a very gradual manner, thus the stretching exponent $\beta$ is small, whereas the ones with large $k$ show a rapid decay in their survivor probability ($\beta$ is large). Since the high$-k$ clusters have a much larger relaxation time than the low$-k$ clusters, see Figure 4 of the main text, we can conclude that for this temperature the former can be considered as basically rigid structures that move in a liquid that relaxes faster.

Panel (c) of Supplementary Figure 5 shows that if temperature is lowered to $T = 950$ K, the $k-$dependence of the cluster lifetime is less pronounced showing that at this temperature the relaxation dynamics becomes again more homogeneous. Note that the length of the simulation runs are independent of temperature. This makes that the statistics of the results for low temperatures is inferior to the one at high temperatures since fewer independent configurations are accessed. On the other side observables that involve, e.g., clusters with high $k$ will not have a good statistic at high $T$ either because at high temperatures there are only a few of them.

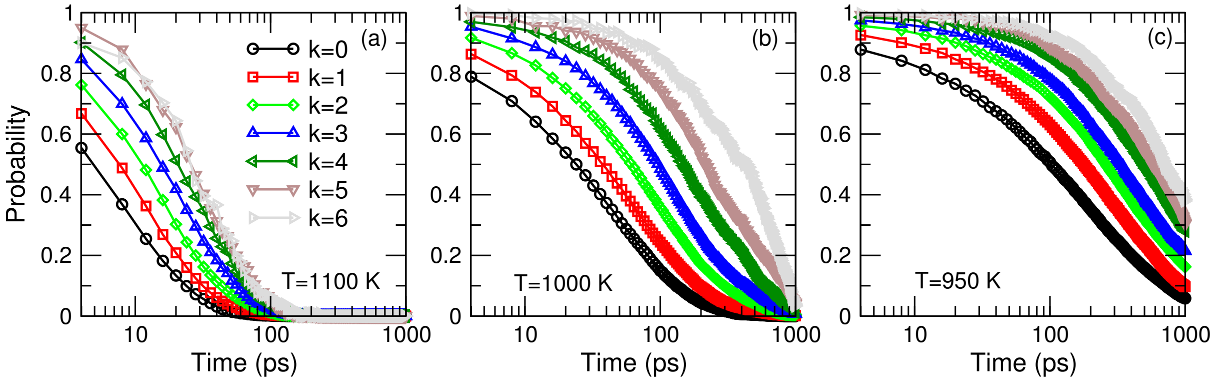


Supplementary Figure 5. Probability that a $k-$cluster that exists at time zero is still intact at time $t$.

**Supplementary Note 6**: Intermediate scattering function and relaxation times

In Supplementary Figure 6 we show the time dependence of the self intermediate scattering function of the Cu atoms for different temperatures. The wave-vector is 2.8 Å$^{-1}$. We see that for $T$ around 1250 K the correlator starts to show a weak shoulder and thus we can identify this with the onset temperature of the system.

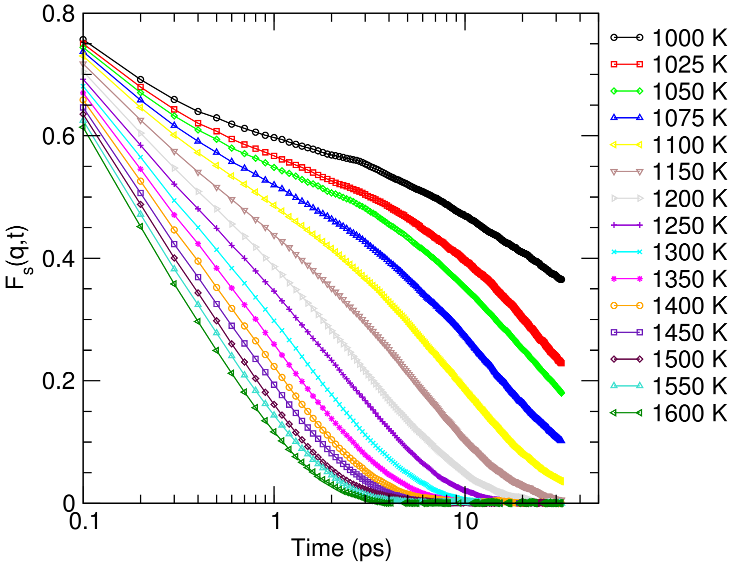


Supplementary Figure 6. Time dependence of the self intermediate scattering function for the Cu atoms at different temperatures. The wave-vector is $q = 2.8$ Å$^{-1}$.

From the correlation functions shown in Supplementary Figure 6 one can obtain the $\alpha$-relaxation time $\tau(T)$ via the definition $F_s(q, \tau) = e^{-1}$. Supplementary Figure 7 is an Arrhenius plot of $\tau$ and it demonstrates that the system has a quite strong non-Arrhenius behavior. Also included in the graph are fits to the data with a Vogel-Fulcher-Tammann law, $\tau(T) \propto \exp[E/(T - T_{\rm VFT})]$, as well as a fit to the data with the power-law proposed by mode-coupling theory, $\tau \propto (T - T_{\rm MCT})^{\gamma}$. At this stage we do not want to put much emphasis on the quality/significance of these fitting functions but we include them in order to obtain a better idea on the relevant temperature scales of the system. From these temperatures we thus can conclude that the change in transport mechanism discussed in the main text occurs at a temperature that is close to $T_{\rm MCT}$, the critical temperature of mode-coupling theory.

In Supplementary Figure 8(a) we show the time dependence of $F_s(q, t)$ at $T = 1000$ K and relatively small wave-vector $q = 0.7$ Å$^{-1}$, i.e., a wave-vector for which the correlator is strongly compressed (see Figure 3 of the main text). In comparison with the data for $q = 2.8$ Å$^{-1}$, shown in Supplementary Figure 8(c), the time correlation function decays significantly slower and is more compressed, in agreement with the discussion of the main text. In Supplementary Figure 8(b) and (d) we show $F_s(q, t)$ for the two other temperatures ($q = 2.8$ Å$^{-1}$) and one sees that i) the relaxation time quickly increases with decreasing temperature, as expected for a glass-forming system; ii) the steepness of the decay in the

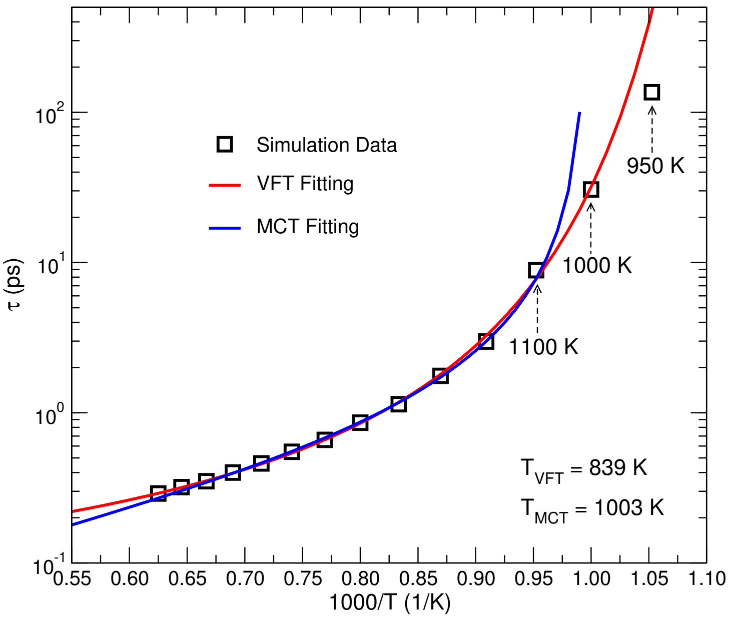


Supplementary Figure 7. Arrhenius plot of the relaxation time as obtained from the self intermediate scattering function for the Cu atoms at wave-vector $q = 2.8$ Å$^{-1}$.

$\alpha-$relaxation regime is largest for $T = 1000$ K, i.e., it is non-monotonic as a function of $T$; iii) the $k-$dependence of the relaxation times is strongest at $T = 1000$ K.

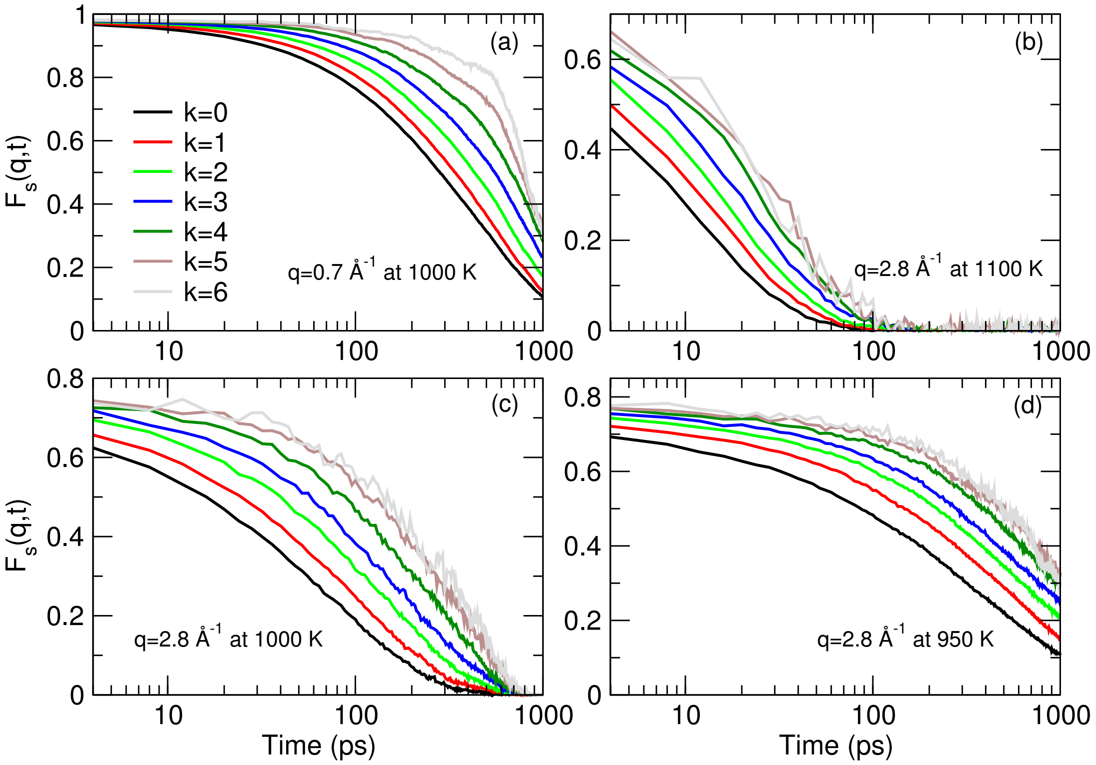


Supplementary Figure 8. Time dependence of the self intermediate scattering function for the different $k-$clusters at $T = 1000$ K with $q = 0.7$ Å$^{-1}$, panel (a), and for $q = 2.8$ Å$^{-1}$ and different temperatures (panels (b)-(d)).

The results discussed in the main text concern mainly the dynamics of the system in its supercooled liquid state. However, one expects that the $k-$dependence of the vibrational features on the sub-picosecond time scale can also be observed in the glass-state. That this is indeed the case is demonstrated in Supplementary Figure 9 where we show the self intermediate scattering function at 300 K for various types of particles. One recognizes that the Cu atoms with high $k$ do indeed show a vibrational motion that is much more pronounced than the one for the Cu with low $k$, in agreement with expectation. This result is also coherent with the $k-$dependence of the vibrational density of states shown in Fig. 2**c** of the main text.

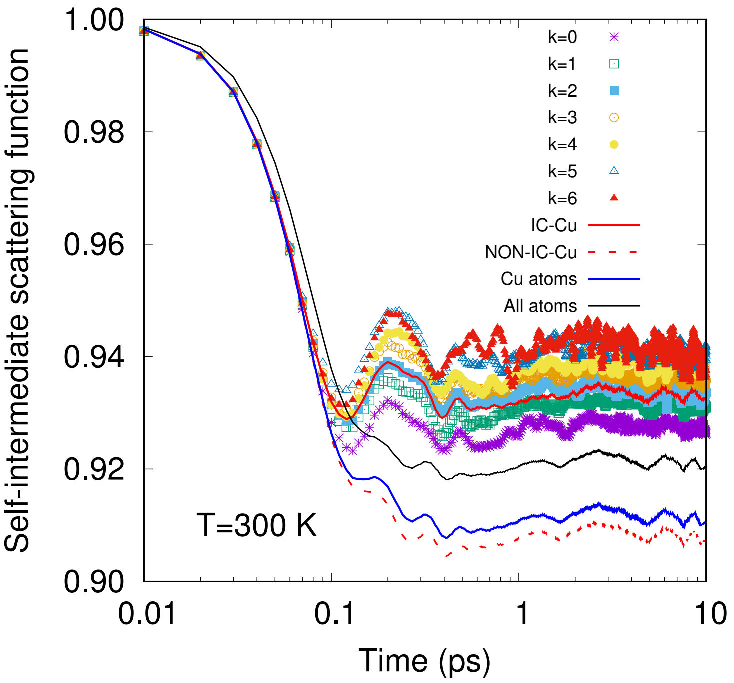


Supplementary Figure 9. Self intermediate scattering function for $q = 2.8$ Å$^{-1}$ at $T = 300$ K. The different curves correspond to different values of $k$. Also included is the data for all the Cu atoms and for all atoms.

## References

[1] Yamamoto, R. & Onuki, A. Heterogeneous Diffusion in Highly Supercooled Liquids. *Phys. Rev. Lett.* **81**, 4915-4918 (1998).
